# Gynecological Problems in Newborns and Infants

**DOI:** 10.3390/jcm10051071

**Published:** 2021-03-04

**Authors:** Katarzyna Wróblewska-Seniuk, Grażyna Jarząbek-Bielecka, Witold Kędzia

**Affiliations:** 1 Department of Newborns’ Infectious Diseases, Chair of Neonatology, Poznan University of Medical Sciences, 60-535 Poznan, Poland; 2 Department of Perinatology and Gynecology, Division of Developmental Gynecology and Sexology, Poznan University of Medical Sciences, 60-535 Poznan, Poland; graja@ump.edu.pl (G.J.-B.); witold.kedzia@ump.edu.pl (W.K.)

**Keywords:** newborn, developmental gynecology, pediatric gynecology, ovarian cysts, atypical-appearing genitals, hydrocolpos

## Abstract

Pediatric-adolescent or developmental gynecology has been separated from general gynecology because of the unique issues that affect the development and anatomy of growing girls and young women. It deals with patients from the neonatal period until maturity. There are not many gynecological problems that can be diagnosed in newborns; however, some are typical of the neonatal period. This paper aims to discuss the most frequent gynecological issues in the neonatal period.

## 1. Introduction

Gynecology (from the Greek word ‘gyne’ = woman) is the area of medicine that specializes in the diagnosis and treatment of diseases affecting female reproductive organs (“woman’s diseases”). In a broader sense, this medical specialty covers the entire woman’s health, including preventive actions, and represents the specificity of anatomical and physiological distinctness of sex. Pediatric-adolescent gynecology or developmental gynecology is separated from general gynecology because of the unique issues that affect the development and anatomy of growing girls and young women. It deals with patients from the neonatal period until maturity. Girls with gynecological disorders constitute 10% of all gynecological patients. That is why they should be included in specialistic gynecological care, particularly in its preventive aspect.

There are not many gynecological problems leading to abnormalities in later development that can be diagnosed in newborns. There are, however, some disorders that are typical of the neonatal period. As far as health promotion and prevention are concerned, it is essential to take care of a baby girl’s health in relation to her future maternity since birth. In the neonatal period, it is crucial to instruct mothers of the proper hygienic procedures for girls’ genitals and of how to prevent diaper dermatitis. Adequate feeding is also important with regard to development.

The neonatologist or the pediatrician is usually the first specialist who detects genital abnormalities in a little girl. In most cases, the related changes are physiological. However, if these abnormalities are disturbing or unusual, the baby should be referred to a pediatric gynecologist for a proper gynecological examination and to a pediatric endocrinologist or pediatric surgeon for further examination and treatment.

Knowledge of normal prepubertal anatomy and the use of an accurate terminology are essential for describing and documenting anatomic findings. The gynecologist assesses the appearance of the vulva, the size and color of labia minora and majora, the introitus of the vagina, the color of the mucous membrane, and the size of the clitoris. It is also essential to evaluate the hymen and the presence of a discharge from the vagina (fluor neonatalis). This examination is mostly performed visually, without the use of instruments. Normally, the labia majora are full, and the labia minora are thickened. The hymenal folds appear thick and redundant. The vaginal mucosa is pink and moist, with an acidic pH. As the maternal hormone levels decrease, the labia majora lose their fullness, and the labia minora and hymen become thinner and flatter. Visualization of the mid and upper vagina may be accomplished while the baby is in the knee-chest position but often requires a sedated examination and instruments. When performing an anesthesia examination, a lighted Killian nasal speculum and a fiberoptic scope (cystoscope, flexible hysteroscope) are useful for examining the prepubertal vagina. A liquid distention medium can be used for vaginoscopy to visualize the whole vagina and cervix. Vaginal cultures should be obtained before the vaginoscopy. The cervix, uterus, and adnexa in the child can be evaluated through an examination, using a finger placed rectally and the other hand abdominally, with the patient lying supine. An ultrasonographic exam allows for assessing the uterine proportions and the appearance of the ovaries. For newborns and small girls, the cooperation between the gynecologist and the mother is obligatory. Educating the mother before this examination is essential both for her reassurance and for gaining her trust.

## 2. Vagina and Hymen

In utero, the vaginal epithelium of the neonate is stimulated by maternal hormones that cross the placenta into the fetal circulation. After delivery, these hormone levels fall rapidly, and a thick, grayish-white, mucoid discharge from the neonate’s vagina can be observed. The secretion usually resolves in 10 days.

In some baby girls, the discharge from the vagina is blood-tinged or even grossly bloody. This is usually treated as physiological menstrual-like neonatal bleeding, and it is assumed that no treatment is needed. However, in some studies, it has been shown that such bleeding might be a marker of intrauterine distress in late pregnancy. It is more often observed in infants with intrauterine growth restriction or in those whose mothers suffered from preeclampsia. Moreover, neonatal menstrual-like bleeding may represent a sign of increased risk of developing endometriosis during adolescence and plays a role in the transgenerational evolution of major reproductive disorders [1,2,3]. Prepubertal bleeding outside the neonatal period is always abnormal and is very alarming to parents and pediatricians. Its cause can be a foreign body, trauma, and vulvovaginitis.

A foreign body is a common cause of prepubertal vaginal bleeding. Most commonly, the object is toilet paper, a small plastic toy, a cap, or other small objects. Bleeding in such a situation might be accompanied by pelvic pain and a foul-smelling discharge. The unidentified foreign body may lead to urinary tract infection or dermatosis and, in serious cases, to perforation into the peritoneal cavity or fistula formation [4].

**Figure 1:**
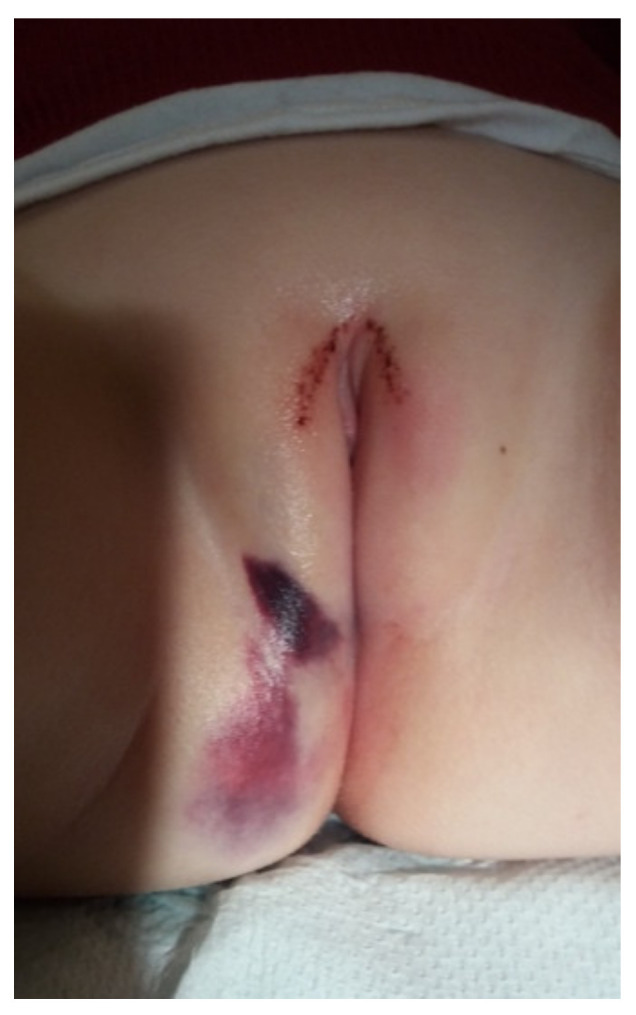
Straddle injury in an infant.

A trauma leading to vaginal bleeding in infants can be either accidental or nonaccidental. The most common injury is the straddle injury following a fall on the edge of some object (Figure 1). It rarely results in deep lacerations and usually spares the hymen and the vagina; ecchymosis and swelling are typical. A nonaccidental trauma with vaginal bleeding may be the result of sexual abuse. When attempting to assess whether the injury was accidental or not, it is essential to consider if the harm matches the story provided by the parents or caregivers [4].

**Figure 2:**
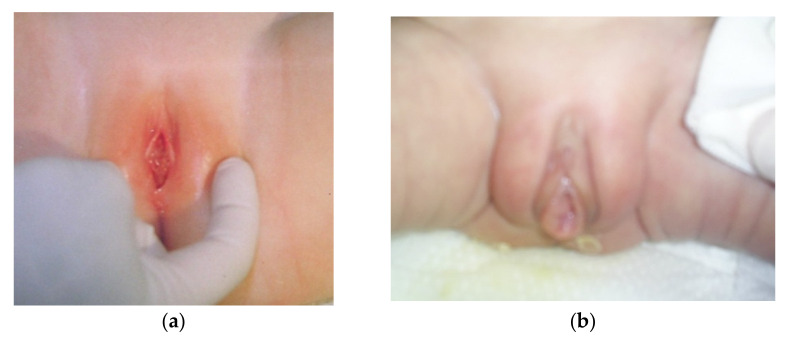
(**a**). Vulvovaginitis in a two-month-old girl. (**b**). Vulvovaginitis in a premature newborn (29 weeks of gestational age).

Vulvovaginitis (Figure 2) is another condition that may lead to vaginal bleeding in infants. The labia minora in young girls are small and do not offer adequate protection to the vaginal opening. Vulvar skin and vaginal mucosa are thin and fragile due to the hypoestrogenic state. In such conditions, even mild insults, such as inflammation or scratching, lead to a breakdown of the epithelium and bleeding. It is known that 75% of cases of vulvovaginitis in infants are nonspecific. Specific vulvovaginitis might be caused by respiratory pathogens (group A *Streptococcus*, *S. pyogenes*), enteric pathogens (*Shigella* spp., *Yersinia* spp.), or *Candida* spp. in infants wearing diapers [4,5].

Other skin conditions predisposing to vaginal bleeding are atopic dermatitis (eczema and psoriasis) and lichen sclerosis [4]. Trauma from rubbing or scratching pruritic areas can lead to prepubertal vaginal bleeding as the presenting symptom of a vulvar dermatologic condition. The etiology of lichen sclerosis is uncertain, but it is generally considered to be at least in part an autoimmune phenomenon. The classic physical examination finding is hypopigmentation in a figure-of-eight pattern, peri-introital and perianal. In the case of atopic dermatitis or eczema, a clinical examination will reveal erythematous skin, which can be scaly at the periphery with evidence of the classic thickened skin of chronic inflammation in the labial creases. Children usually have other similar lesions on the body, as well as a history of other allergies [4,5].

Vaginal bleeding may also be a sign of a gynecologic tumor, such as ovarian, vaginal, or vulvar tumor. The most common malignant tumor in young girls is rhabdomyosarcoma, specifically, sarcoma botryoides. The peak age for the development of this tumor is two years, and over 90% of them are detected before five years of age. Patients may present with an intralabial mass that is grape-like in appearance and vesicular [4]. Endodermal sinus tumors are germ-cell in origin and found primarily in infants younger than two years. The most common symptoms are vaginal bleeding and discharge, and a polypoid or sessile tumor usually originates from the posterior fornix [4,5].

In newborns, the hymen is redundant, estrogenized, thick, and elastic, often with a prominent ridge at six o’clock. As the estrogen subsides, the hymen becomes unestrogenized, thin, and significantly more vascular. Assessment of the hymen includes the observation of its shape, variations in the hymenal rim, the degree of estrogen effect, and any signs of trauma or scar tissue. The examination might reveal anatomic variants that sometimes are mistaken for signs of sexual abuse, such as midline sparing (linea vestibularis), failure of midline fusion, urethral prolapse, or labial adhesion. Midline sparing refers to a symmetric, flat avascular area of the posterior vestibule observed in 10% of healthy newborns. It can sometimes be confused with scarring. Congenital abnormalities of the hymen include imperforate, microperforated, cribriform, septated, and annular hymens. They occur in 3–4% of the female population. Redundant or fimbriated hymens that appear to be sleeve-like or ruffled are common in newborns because of maternal estrogen effects. The crescentic shape becomes the most common shape around the age of 4 years in the prepubertal female [5]. Typical findings on the hymen that are often mistaken for pathology include hymeneal tags, mounds, or bumps on the rim, as well as notches or clefts in the anterior portion of the hymen—above the three- and nine-o’clock locations. On the contrary, healed hymeneal transections or the absence of hymeneal tissue extending to the base in the posterior half of the hymen are indicative of trauma and may signify sexual abuse of the child. Healed deep notches at or below the three- to nine-o’clock position, more profound than a superficial notch and extending nearly to the base of the hymen, but not a complete transection, should be interpreted with caution, as they are not consistent with sexual trauma [5].

In the presence of congenital malformations such as vaginal atresia or an imperforate hymen, the physiological discharge of the vaginal epithelium stays inside or releases in drops only when the baby cries. The incidence of imperforate hymen is estimated at 0.014–0.1%, and its presentation is often late, with amenorrhea in adolescence [6,7]. However, in some patients, it might present in the neonatal period as hydrocolpos, which is a cystic dilation of the vagina with fluid accumulation, due to the stimulation of secretory glands of the reproductive tract secondary to vaginal obstruction [6]. It presents as a soft mass protruding from the external genitals. In severe cases, the imperforate hymen and hydrocolpos may result in renal complications in the neonatal period. They can lead to obstructive uropathy through the compression of the lower urinary tract, resulting in hydronephrosis and hydroureters [6]. Early detection is essential, as this malformation could be associated with life-threatening renal failure. There have been rare cases of familial occurrence of imperforate hymen [8]. Most cases, however, are thought to occur sporadically, and no genetic mutations have been identified [6,8].

Prenatal diagnosis of imperforate hymen with hydrometrocolpos has been reported as early as 25 weeks of gestation, although most cases are described during late pregnancy or after birth [7,8,9,10]. Imperforate hymen accounts for 15% of abdominal masses in female infants. The differential diagnosis of such pelvic mass should include distended urinary bladder, ovarian neoplasm, reduplication of the sigmoid, and sacrococcygeal teratoma. Associated genitourinary and anorectal anomalies have been described, such as persistent urogenital sinus, nephronia, or cloacal dysgenesis [7,8,9,10]. Asymptomatic imperforate hymen can be managed without early surgical intervention. However, if the imperforate hymen causes obstructive symptoms due to hydrometrocolpos, then hymenectomy is indicated [11]. 

Labial adhesions are identified when the labia minora are partly or entirely agglutinated. The incidence is reported to be around 1.8%, and the diagnosis is done most frequently between 13 and 23 months of age when the estrogen level is physiologically low [12]. The symptoms might be related to urinary outlet obstruction, but more than 35% of labial adhesions are reported to be asymptomatic. Sometimes, urogenital infections, pain in the genital area, and problems with urination may be observed. A topical estrogen ointment applied to the adhesion area is often used as a first-line treatment option, and manual separation of the labia is reported as a second-line treatment when topical treatment fails. Treatment is usually recommended only in symptomatic cases [12].

Interlabial cysts are another abnormality that may cause anxiety to the parents and pediatricians. They occur in 1:1000 to 1:7000 newborn girls. Most commonly, they are simple hymenal cysts (labial cysts) and paraurethral gland cysts (epithelial inclusion or Skene cysts) and present as single small, bulging, golden-colored, or whitish interlabial protrusion adjacent to the urethral meatus and slightly distorting it. The cysts usually resolve spontaneously over 2–3 months, and their presence is related to exposure to maternal estrogens. The differential diagnosis of interlabial masses includes anomalies of the ureter or urethra, ectopic tissue, prolapse (prolapsed urethra, ureterocele, vagina, or uterus), or neoplasia (botryoid rhabdomyosarcoma) [13].

## 3. Mammary Glands

An essential part of the physical examination of newborns is an assessment of mammary glands. Breast development occurs during fetal life and is well described as proceeding in female infants for several months after birth. It ceases at 3–6 months of age, and the breast bud may regress after that [14].

Neonatal breast enlargement is defined as the benign proliferation of glandular tissue due to estrogens crossing the placenta into the fetal circulation. It occurs in approximately 70% of newborns, both girls and boys, and can be unilateral or bilateral. Usually, the diameter of a breast bud is 1 to 2 cm in the first few weeks of life. Mammary glands in newborns are often hard, hyperemic, and tender. In some newborns, the breasts are excessively big, and the reason for such an exaggerated response to maternal hormones is unclear [14,15].

Postnatally, falling levels of maternal estrogens are thought to trigger prolactin secretion in the pituitary of the newborn. Prolactinemia stimulates neonatal breasts and causes milk secretion in 5 to 20% of newborns [14,15,16]. Milk that comes from the breast of a newborn baby is called “witch’s milk” [16]. It resembles maternal milk, with an identical concentration of IgA, IgG, lactoferrin, lysosome, and lactoalbumin [17]. The nipple discharge can also be bloody at times, which is due to mammary duct ectasia [14]. The ability of a baby’s breasts to respond in this fashion is an indication that the baby was born at or near full term. The breast of a baby born prematurely cannot enlarge or produce milk. It is interesting that milk secretion is more common in the presence of neonatal hypothyroidism [18,19,20]. In babies with this disease, focal breast uptake of 99mTc-pertechnetate during a thyroid scan may be observed even without clinically palpable breast nodules on either side [18,19].

When the mammary glands are enlarged and produce milk, the infant may be more susceptible to breast inflammation and infection. Inadequate secretion of milk due to improper canalization of the lactiferous ducts may lead to stagnation of milk (galactocele). In such a situation, the infection may result in complications such as mastitis and breast abscess. Neonatal mastitis usually occurs under five weeks of age, with a peak incidence at three weeks of age, and is mostly unilateral. The most common causative agent is *Staphylococcus aureus* (83–88%). Typical presentation includes unilateral swelling, erythema, warmth, tenderness, and induration in the absence of systemic signs of infection. If left untreated, it can lead to breast abscess and, rarely, to generalized sepsis [14,15,21].

Supernumerary nipples are a common finding. They might extend along the nipple line on either side of the chest wall, down the abdomen, and may occur in the labia. Bilateral ectopic breast tissue has been described in the vulva [22].

## 4. Atypical-Appearing Genitals

Atypical-appearing genitals pose significant neonatological, gynecological, genetic, surgical, and psychological problems. The examination of the external genitals should include precise measurements of anatomic features, in reference to the normative values [23,24,25]. The Prader scale initially developed for congenital adrenal hyperplasia (CAH) is a scoring system for grading genital masculinization [26,27,28,29]. Grade 0 refers to an unvirilized female, while grade 5 describes a completely virilized female—a female who appears externally male at birth but with the labial/scrotal sac empty, since it contains no testicles [27]. Prader virilization stages are presented in Table 1.

Drs. Charmian Quigley and Frank French proposed a scale to grade the appearance of external genitalia in babies with androgen insensitivity syndrome (AIS), as shown in Table 2. Grades 6/7 reflect complete androgen insensitivity (CAIS), and the sex of rearing is invariably female. In babies with partial androgen insensitivity (PAIS), the genitals may appear almost entirely feminine (Grade 5), mixed male/female (Grade 2–4), or altogether male (Grade 1). Some babies with PAIS may be raised as males, but many are re-assigned as females. Slight androgen insensitivity might contribute to infertility in some otherwise normal men [30].

The diagnosis and treatment of a child with disorders of sex development (DSD) require a multispecialty team experienced in the long-term management of such patients and generally available only in tertiary-care centers. An integrated approach is essential to convey consistent information and an appropriate plan to the parents. They should be given a complete biological background concerning sex determination and the potential causes of DSD in their child, presented in a manner that ensures their understanding of the issues. Parents should be kept fully informed regarding planned diagnostic tests and the interpretation of results. Psychological counseling should be offered to the parents on how to optimally share critical information with more distant members of their family. If surgical intervention is recommended, indications, timing, and access to appropriate services should be discussed with the parents.

While our understanding of the genetics of sex determination has advanced rapidly (more than 30 genes are known to contribute to the etiopathogenesis of DSD), the specific molecular disorder may not be defined in many affected children. Existing algorithms are of limited value, due to the broad spectrum of phenotypes and genetic polymorphisms as well as the often long time needed to complete genotyping in highly specialized, but rare, laboratories with appropriate expertise [23,26,28]. Prenatal and family history, as well as physical examination, represent the essential first step in the diagnosis of DSD.

Physical examination includes inspection of the neonate for potential dysmorphic features, including head or facial dysmorphisms, orthopedic or heart defects, as DSD may be part of congenital syndromes. The presence of a micropenis due to hypogonadotropic hypogonadism may suggest a coexistent pituitary dysfunction with secondary hypocortisolemia and hypothyroidism. The examination of the external genitalia includes the precise measurement of anatomical structures, in relation to normative values. Besides, the analysis includes the detecton of the presence of gonads, hyperpigmentation of the external genitalia, breast areola and ear lobes, crease of the labioscrotal folds, length, width, and symmetry of the corpora, ventral flexion of the penis, presence and degree of hypospadias, degree of labioscrotal fusion. A well-formed penis and significant labioscrotal rugae may suggest prenatal exposure to elevated testosterone and dihydrotestosterone (DHT) levels. Asymmetric virilization indicates that only one gonad secretes testosterone. A contralateral dysgenetic testis or ovary is present (in the case of ovotesticular DSD) [31,32,33].

## 5. Ovarian Cysts

**Figure 3:**
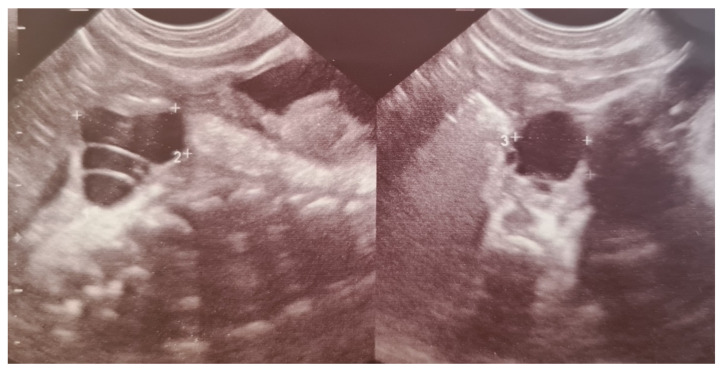
Simple and complex ovarian cysts in a premature newborn girl. The complex cyst contains septa.

Ovarian cysts usually arise from mature follicles and are the most common abdominal cysts in female fetuses and newborn girls [34,35]. They result from fetal exposure to maternal and fetal gonadotropins and are found more often in newborns whose mothers had increased levels of human chorionic gonadotropin – hCG (diabetes, maternal isoimmunization). Most of them are detected prenatally [34,35]. Ultrasonography is the imaging technique of choice for diagnosing ovarian cysts, as it makes it possible to differentiate them from other cystic lesions. Most ovarian cysts are functional, benign follicular cysts that do not interfere with the course of pregnancy or birth. They can be divided into simple or uncomplicated—completely anechoic, homogenous, and thin-walled—and complex or complicated—characterized by fluid-debris level and a thick echogenic wall, which may contain solid components, clot, or septa (Figure 3). In infants, complex cysts are most consistent with ovarian torsion that occurred prenatally. Frequently, even complex cysts regress after birth, as maternal hormone stimulation decreases [35,36,37].

Torsion is the most common complication of ovarian cysts (50–78%). It appears antenatal in most cases and is more common in large cysts. This complication may cause inflammatory adhesions, intracystic bleeding, and rupture. In large cysts, pulmonary hypoplasia or polyhydramnios have been described due to the pressure of the mass on the small intestine, interfering with the fetal swallowing mechanism [35].

The management of congenital ovarian cysts is controversial. It depends on cyst size and complications that might occur during gestation or after birth. Early surgical intervention used to be recommended to prevent torsion and cyst hemorrhage or rupture that may lead to intestinal obstruction. The current trend favors watchful waiting and close follow-up with serial ultrasounds until cyst involution, especially for cysts smaller than 4 cm, which are less likely to be associated with torsion [38,39]. These cysts may regress by the end of the first year of life, but others may persist and require surgical intervention [39]. On surgery, the recommendation is to preserve the ovary unless the pathology is likely to be malignant [39,40]. Aspiration of simple ovarian cysts, either percutaneously or laparoscopically, is a minimally invasive alternative to resection [41,42,43]. Such management also reduces the risk of torsion and preserves the ovarian tissue.

Symptomatic cysts and those that do not regress after several months should be resected. Complex masses that can lead to such problems as cyst recurrence and small bowel obstruction also require surgical management [44]. Concern for malignancy is another indication of surgery for large, complex cysts, mainly if solid components or calcifications are present on imaging. Tumor markers, such as alpha-fetoprotein (AFP) and beta-hCG, may help guide the decision to operate rather than observing. However, their elevation in neonatal patients and infants is of unclear significance. In a review of 170 antenatally diagnosed ovarian cysts, two teratomas and three cystadenomas were identified, and the overall malignancy rate was low [38].

In preterm babies, multiple ovarian cysts may result from excessive ovarian stimulation syndrome [45]. Other signs are edema in the vulva, hypogastric area, and thigh as well as breast enlargement. Solitary or monthly vaginal bleeding may occur in cases with a severe clinical course [45]. The pathophysiology of this syndrome is not fully known. Still, it is thought that lack of maturation of the hypothalamo–pituitary–gonadal axis and lack of negative feedback mechanisms with discontinuation of placental steroids are involved.

## 6. Conclusions

Gynecological problems in neonatal life are unusual and rare. Many conditions that are thought to be pathological are, in fact, anatomical variants or physiological situations. In some cases, however, it is necessary to monitor the newborns with gynecological problems over a longer time to promote fertility and supervise the prepubertal phase. Therefore, knowledge of these abnormalities is essential so that explanations and prognosis can be reassuringly offered to parents.

## Figures and Tables

**Table 1:** Prader Virilization Stages for Genital Masculinization [27]. NF—normal female; NM—normal male

NF	unvirilized female
I	mild clitoromegaly without labial fusion, separate vaginal and urethral orifices
II	clitoromegaly and posterior labial fusion
III	greater degree of clitoromegaly, single perineal urogenital orifice, and almost complete labial fusion
IV	increasingly phallic clitoris, urethra-like urogenital sinus at the base of the clitoris, and complete labial fusion
V	penile clitoris, urethral meatus at the tip of the phallus, and scrotum-like labia (appear like males without palpable gonads)—completely virilized female
NM	normal male presentation, normal testes

**Table 2:** Quigley–French Scale for androgen insensitivity syndrome (AIS) [30]. PAIS, partial androgen insensitivity, CAIS, complete androgen insensitivity.

Grade 1	Mild AIS (MAIS)	Male Genitals, Infertility
Grade 2	PAIS	male genitals but mildly ‘under-masculinized’, isolated hypospadias
Grade 3	PAIS	predominantly male genitals but more severely ‘under-masculinized’ (perineal hypospadias, small penis, cryptorchidism, i.e., undescended testes, and/or bifid scrotum)
Grade 4	PAIS	ambiguous genitals, severely ‘under-masculinized’ (a phallic structure that is indeterminate between a penis and a clitoris)
Grade 5	PAIS	essentially female genitals (including separate urethral and vaginal orifices, mild clitoromegaly, i.e., enlarged clitoris)
Grade 6	CAIS	female genitals with pubic/underarm hair
Grade 7	CAIS	female genitals
